# The role of response modalities in cognitive task representations

**DOI:** 10.2478/v10053-008-0085-1

**Published:** 2011-07-20

**Authors:** Andrea M. Philipp, Iring Koch

**Affiliations:** 1 Department of Psychology, RWTHTH Aachen University, Aachen, Germany; 2 Department of Psychology, Max Planck Institute for Human Cognitive and Brain Sciences, Leipzig, Germany

**Keywords:** response modalities, cognitive control, motor control, task switching, task representation

## Abstract

The execution of a task necessitates the use of a specific response modality. We examined the role of different response modalities by using a task-switching paradigm. In Experiment 1, subjects switched between two numerical judgments, whereas response modality (vocal vs. manual vs. foot responses) was manipulated between groups. We found judgment-shift costs in each group, that is irrespective of the response modality. In Experiment 2, subjects switched between response modalities (vocal vs. manual, vocal vs. foot, or manual vs. foot). We observed response-modality shift costs that were comparable in all groups. In sum, the experiments suggest that the response modality (combination) does not affect switching per se. Yet, modality-shift costs occur when subjects switch between response modalities. Thus, we suppose that modality-shift costs are not due to a purely motor-related mechanisms but rather emerge from a general switching process. Consequently, the response modality has to be considered as a cognitive component in models of task switching.

## Introduction

In cognitive psychology, it is usually assumed that, for each task, there exists a cognitive representation of processes necessary to perform this task. This cognitive task representation is termed *task set* (see e.g., Rogers & Monsell, 1995; Vandierendonck, Christiaens, & Liefooghe, 2008). For example, to perform a parity task (“Is a digit odd or even?”), subjects are assumed to encode a visually presented stimulus, to decide whether it is odd or even, and to indicate this decision by pressing one of two response keys. The notion of *cognitive task representations* is often centered on cognitive aspects of a task. That is, for example, the numerical judgment or the mapping of a stimulus category (e.g., “odd”) to a response category (e.g., “left”). In contrast to these cognitive aspects, the role of the motor execution has received only very little research attention. Specifically, it is widely unknown whether the modality (e.g., vocal vs. manual) in which a response is executed affects task performance and the cognitive representation of tasks.

In the present study, we aimed to investigate the role of the response modality in cognitive task representations. To do so, we used a task-switching paradigm (for reviews, see e.g., Kiesel et al., 2010; Monsell, 2003). In the task-switching paradigm, subjects are introduced to different tasks and are required to execute them in a changing sequence. When subjects have to switch the task from one trial to the next, performance is usually worse than when a task is repeated in two successive trials (“shift costs”, see e.g., Allport, Styles, & Hsieh, 1994; Meiran, 1996; Rogers & Monsell, 1995). In the cuing version of the task-switching paradigm (see Meiran, 1996), the relevant task of each trial is indicated by a cue. By manipulating the time between cue and imperative stimulus (cue-stimulus interval, CSI), one can also manipulate the task preparation time.

 To explore the role of response modalities (i.e., the influence of motor execution) in task switching we specifically examined which effects are observed when subjects switch between response modalities. However, before we turn to this question, it is also important to ask whether task-switching performance is affected by the response modality subjects are required to use (e.g., vocal vs. manual responses throughout the experiment). Some findings indicated the relevance of this question. For example, Hunt and Klein (2002) compared eye movements and manual responses in a task-switching experiment. In their study, one group of subjects performed eye-movements towards or away from a visually presented stimulus (i.e., prosaccades vs. antisaccades), and another group of subjects performed spatially compa-tible or incompatible key presses to the same kind of stimuli. The result pattern differed between both response modalities: Residual shift costs (i.e., that part of shift costs that remains even after a long CSI; Rogers & Monsell, 1995) were found for manual responses but not for eye-movements. In another study, Brass and von Cramon (2007) compared manual and foot responses in a task-switching study. In this study, subjects were required to switch between two numerical judgments (magnitude vs. parity). In one half of the blocks, subjects responded manually, in the other half they responded by foot responses. With respect to shift costs both the behavioral data pattern and the prefrontal brain activation observed with functional magnetic resonance imaging (fMRI) were comparable for manual and foot responses. Yet, a difference emerged again with respect to the influence of preparation time. After a long preparation time, residual shift costs were largely eliminated for manual responses but not for foot responses. 

 Furthermore, the influence of the response modality appears to depend on specific aspects of the task set, for example the stimulus modality (e.g., Stelzel, Schumacher, Schubert, & D’Esposito, 2006). Recently, Stephan and Koch (2010) demonstrated that both reaction time (RT) and shift costs increase when incompatible stimulus modality/response modality pairings (i.e., visual-vocal and auditory-manual) are used as compared to compatible stimulus modality/response modality pairings (i.e., visual-manual and auditory-vocal). 

In the present study (Experiment 1), we compared task-switching performance across three different response modalities: vocal, manual, and foot responses. In Experiment 2, we were specifically interested in the effects of switching between different response modalities. Here, previous studies (Arrington, Altmann, & Carr, 2003; Sohn & Anderson, 2003; Yeung & Monsell, 2003) suggest that a switch of the response modality increases shift costs. For example, in the study of Arrington et al., subjects switched among four different tasks. Each task had its own response categories; two tasks required vocal responses and two tasks required manual responses. The results showed that the shift costs were larger when both the response modality and the response categories had to be switched as compared to switching the response category only. However, all studies mentioned above do not allow examining the effect of response modalities or motor execution in isolation (i.e., independent from the influence of the cognitive control processes necessary to switch between response categories or judgments). This argument also applies to a study of Philipp and Koch (2010), in which subjects switched between two numerical judgments and two response modalities. 

 Thus, in Experiment 2 of the present study, subjects had to perform the same numerical judgment in each trial but switched between response modalities (e.g., vocal and manual responses). A similar procedure was already adopted in the study of Philipp and Koch (2005). However, as regards that study, it is important to note that each subject switched among three response modalities (i.e., vocal vs. manual vs. foot responses) and a repetition of response modalities was excluded so that questions concerning modality-shift costs could not be addressed. In contrast, the present study focused explicitly on modality-shift costs and their possible reduction with a long preparation time.

The observation of modality-shift costs would improve our understanding of both the mechanisms underlying task switching and the relevance of motor control processes. On the one hand, modality-shift costs would indicate that response modalities can play a role in task switching and, thus, have to be included in models of task switching. On the other hand, modality-shift costs would also indicate that motor control and the selection of a response modality has the same consequences as cognitive control and the selection of a stimulus categorization rule (i.e., judgment).

## Experiment 1

In Experiment 1, we compared three different response modalities: vocal, manual, and foot responses. To this end, subjects switched between two numerical judgment tasks (parity vs. magnitude), whereas the response modality was manipulated between groups (vocal group vs. manual group vs. foot group). Additionally, we manipulated the preparation time (CSI) within subjects to examine preparation effects. The aim of this first experiment was to compare the pattern of results of an identical judgment-switching design across different response modalities.

### Method

#### Subjects, stimuli, and tasks

Twenty-four subjects (13 female, mean age = 26.9 years) were evenly assigned to the three experimental groups (vocal, manual, and foot). They received 8 € for participation. Stimuli consisted of the digits 1-9, excluding 5. Subjects had to decide whether a digit was smaller or larger than 5 (magnitude judgment) and whether it was odd or even (parity judgment). Stimuli were presented one at a time in white in a frame at the centre of a black screen (15“ monitor) connected to an IBM-compatible PC. The digits were 1 cm high and approximately 0.5 cm wide. The viewing distance was approximately 60 cm. The frame, which served as judgment cue, was white and had either the shape of a diamond (5.3 cm wide/high), indicating the magnitude judgment, or of a square (3.8 cm wide/high), indicating the parity judgment.

Manual responses were made on an external keyboard with two response keys for the left and right index finger. Response keys mea-sured 1.2 cm by 1.7 cm and were separated by 3.8 cm. Foot responses were given on a separate external keyboard with two response keys (6.0 cm by 6.0 cm, separated by 23.5 cm) for the left and right foot. Vocal responses were expressed by saying “left” or “right” (i.e., subjects had to say the German words “links” and “rechts”). Speech onset was recorded in milliseconds using a voice-key; accuracy of “left” and “right” responses were coded by the experimenter with the left and right cursor key.

#### Procedure

The experiment was run in a single session of approximately 60 min. Instructions were both given on the monitor and orally. An instruction sheet concerning the stimulus-response mapping (e.g., “odd-left”) was placed in front of each subject throughout the experiment. The four possible mappings were counterbalanced across subjects.

Two practice blocks were run with ten trials each. One practice block had short CSI (100 ms), the other long CSI (1000 ms). The experiment itself consisted of eight blocks of 96 trials each. Before each block, subjects were informed about CSI in the next block. Blocks with short and long CSI alternated within one experiment; CSI duration in the first block was counterbalanced across subjects.

A trial started with a black screen followed by a cue. After a variable preparation time (CSI), the stimulus was presented in the middle of the cue frame and a 600 Hz warning tone was played simultaneously. The interval between the response of the subject and the next stimulus (response-stimulus interval, RSI) was 1.6 s after manual and foot responses. The RSI after vocal responses varied somewhat around 1.6 s because the experimenter had to code vocal responses for accuracy, so that the time between the response of a subject and the next cue (response-cue interval, RCI) depended on the RT of the experimenter. The timing of the vocal trials was such that the interval between response of the subject and cue was identical to the RCI in manual and foot trials – at least when the RT of the experimenter in vocal trials was 300 ms. Thus, in manual and foot trials RSI was held constant at 1600 ms with response-cue interval being either 1500 ms or 600 ms, and CSI being either 100 ms or 1000 ms (i.e., RCI 1500/CSI 100 vs. RCI 600/CSI 1000). Vocal trials had approximately the same RCI and RSI.

The sequence of trials was controlled for an equal number of each numerical judgment, stimulus category (odd vs. even, or smaller vs. larger), and judgment sequence (judgment repetition vs. judgment switch). Immediate repetition of a stimulus was excluded.

Subjects always received visual error feedback for 500 ms when they pressed the wrong key (German: “Falsche Taste”).

#### Design

Judgment transition (judgment switch vs. judgment repetition) and CSI (100 ms vs. 1000 ms) were within-subject independent var-iables when analyzing each group individually in a first step. For the comparison of groups, Group (vocal vs. manual vs. foot) was added as between-subjects variable. RTs and error percentage were measured as dependent variables. For these and all following analyses, significance was tested at α = .05.

### Results

The first two trials of each block were discarded from analysis. Additionally, trials in which the RT was below 200 ms or three stan-dard deviations above each subjects mean RT were discarded from both RT and error analyses (1.8% of the trials). RT analyses included only correct trials preceded by at least one other correct trial. RT and error data are shown in Table 1.

**Table 1.** Experiment 1: Reaction Times as a Function of Judgment Transition (Switch vs. Repetition), Response-Modality Group (Vocal vs. Manual vs. Foot), and Cue-Stimulus Interval (100 ms vs. 1000 ms).

	Judgement transition		
	Switch	Repetition	Shift costs (switch - repeat)
Vocal group			
CSI 100	1187 (5,1)	1031 (2,8)	156 (2,3)
CSI 1000	1002 (5,0)	882 (3,0)	120 (2,0)
Manual group			
CSI 100	815 (7,3)	707 (4,9)	108 (2,4)
CSI 1000	619 (6,3)	548 (4,1)	71 (2,2)
Foot group			
CSI 100	1291 (8,7)	1136 (6,3)	155 (2,4)
CSI 1000	1130 (6,4)	962 (5,2)	168 (1,2)

*Note*. Reaction times in milliseconds. Error percentage in parentheses. CSI = cue stimulus interval.

#### Individual analyses of response-modality groups

In a first step, each group was analyzed separately using two-way analyses of variance (ANOVAs) with judgment transition (judgment switch vs. judgment repetition) and CSI (100 ms vs. 1000 ms) as within-subject independent variables.

For the vocal group, the RT analysis revealed significant effects of judgment transition, *F*(1, 7) = 41.7, *p* < .001, η_p_² = .856; and of CSI, *F*(1, 7) = 16.2, *p* < .01, η_p_² = .698. The interaction of judgment transition and CSI was not significant, *F*(1, 7) = 2.1, *p* = .19. In the error analysis, no main effect or interaction was significant: judgment transition, *F*(1, 7) = 3.9; CSI and judgment transition x CSI, *F*s < 1.

For the finger group, both RT and error analyses revealed a significant effect of judgment transition, *F*(1, 7) = 14.6, *p* < .01, η_p_² = .676 for RT; and *F*(1, 7) = 14.1, *p* < .01, η_p_² = .668 for error data. The effect of CSI was significant in the RT analysis, *F*(1, 7) = 45.7, *p* < .001, η_p_² = .867; but not in the error analysis, *F*(1, 7) = 1.2, *p* = .32. Further, the interaction of judgment transition and CSI was marginally significant in the RT analysis, *F*(1, 7) = 4.8, *p* = .064, η_p_² = .407; but not in the error analysis, *F* < 1.

For the foot group, again the main effects of judgment transition, *F*(1, 7) = 21.8, *p* < .01, η_p_² = .757; and of CSI, *F*(1, 7) = 12.8, *p* < .01, η_p_² = .647, were significant. The error analysis showed a similar pattern: judgment transition, *F*(1, 7) = 4.3, *p* = .076, η_p_² = .382; and CSI, *F*(1, 7) = 14.1, *p* < .01, η_p_² = .669. The interaction of judgment transition and CSI was not significant in either RT or error analysis, *F*s < 1.2.

Taken together, substantial judgment-shift costs as well as a reduction of the overall RT with a long preparation time were observed in all response-modality groups. Thus, judgment-shift costs and a general preparation effect emerged irrespective of the response modality that was used to indicate the response. Yet, differences between groups seem to appear when we look at the reduction of shift costs with a long preparation time. Whereas a numerical (albeit not significant) reduction occurred in the vocal and the finger group, the effect was numerically even reversed in the foot group.

#### Comparison of groups

To directly compare the groups, we further calculated between-group comparisons on RT and error data. Replicating the individual analyses, both RT and error analyses showed significant effects of judgment transition, RT: *F*(1, 21) = 68.7, *p* < .001, η_p_² = .766; and error: *F*(1, 21) = 16.9, *p* < .001, η_p_² = .446; and of CSI, RT: *F*(1, 21) = 57.2, *p* < .001, η_p_² = .731; and error: *F*(1, 21) = 4.5, *p* < .05, η_p_² = .176; but no significant interaction of judgment transition and CSI (*F*s < 1). Group did not produce a significant interaction with judgment transition or CSI in either RT or error analysis. Further, the three-way interaction was not significant (all *F*s < 1.9). Thus, the comparison of groups did not reveal a significant difference between groups with respect to judgment-shift costs or preparation effects.

However, there was a general difference between groups. In the RT analysis, the effect of group was significant, *F*(2, 21) = 5.1, *p* < .05, η_p_² = .325. The RT was 1026 ms for vocal responses, 672 ms for manual responses, and 1130 ms for foot responses. In the error analysis, the main effect of group was also significant, *F*(2, 21) = 3.8, p < .05, η_p_² = .267. Subjects made 3.9% errors in the vocal group, 5.6% in the manual group, and 6.6% in the foot group.

### Discussion

All three response-modality groups showed a comparable data pattern in which a judgment repetition was faster and more accurate than a judgment switch. Thus, judgment-shift costs emerged irrespective of the response modality that was used to indicate a response. Moreover, we found the same general benefit of a long preparation time across all groups. Consequently, we suggest that the response modality had no influence on general judgment preparation effects.

As regards residual shift costs, however, the judgment-shift costs were not reduced with a long preparation time. Furthermore, as the influence of preparation time on the size of shift costs was numerically different between response modalities, the present results could be seen a further indication that residual shift costs may differ as a function of the response modality (see also Brass & von Cramon, 2007; Hunt & Klein, 2002). Yet, at the moment it is difficult to draw any conclusions with respect to the role of task preparation in response modality effects so that further research is needed.

However, for the present purpose it is most important that the modality in which the subjects had to indicate their response decision (i.e., right vs. left) does not significantly influence the general data pattern and the occurrence of judgment-shift costs. Yet, differences were found between the modalities concerning the overall speed and accuracy of responses. This difference presumably resulted from every-day life experiences of subjects, because foot responses are not as well practiced as either manual or vocal responses. Further, we also observed a speed-accuracy trade off between vocal and manual responses. Vocal responses were slower but more accurate than manual responses (cf. Zirngibl & Koch, 2002).

## Experiment 2

In Experiment 2, we examined the effect of switching between response modalities. Here, subjects switched between two different response modalities while the numerical judgment was the same in each trial. To increase the generality, three different response-modality groups were compared. One group of subjects switched between vocal and manual responses (vocal/manual group), one group switched between vocal and foot responses (vocal/foot group), and a third group switched between manual and foot responses (manual/foot group). Additionally, each group was divided, so that half of each group had to perform the magnitude judgment and half had to perform the parity judgment throughout the experiment. However, no systematic effect of the type of judgment was expected (see Philipp & Koch, 2005). Therefore, the type of judgment was not considered in the analysis. Like in Experiment 1, a manipulation of the cue-stimulus interval (100 ms vs. 1000 ms) was included to examine the influence of the preparation time on possible modality-shift

costs.

## Method

Twenty-four new subjects (20 female, mean age = 26.7 years) were tested and received 8 € for participation. They were evenly assigned to the experimental groups. Stimuli, numerical judgments, and procedure were identical to Experiment 1. However, each subject had to perform only one numerical judgment throughout the experiment. Also, the cue frame served as modality cue. In the vocal/manual group, vocal responses were indicated by the diamond and manual responses by the square. In the vocal/foot group, a diamond indicated vocal responses and a square indicated foot responses. Finally, in the manual/foot group, the diamond indicated manual responses and the square indicated foot responses.

In Experiment 2, modality transition (modality switch vs. modality repetition) and CSI (100 ms vs. 1000 ms) were within-subjects variables. Group (vocal/manual vs. vocal/foot vs. manual/foot) was a between-subjects variable. RTs and error percentage were measured as dependent variables.

## Results

The first two trials of each block as well as trials with an RT below 200 ms or three standard deviations above each subjects mean RT were discarded from the analyses (1.8% of the trials). RT analysis included only correct trials preceded by at least one other correct trial.

### RT analysis

The three-way ANOVA with modality transition (modality switch vs. modality repetition) and CSI (100 ms vs. 1000 ms) as within-subject independent variables and with group (vocal/manual vs. vocal/foot vs. manual/foot) as between-subjects variable revealed a significant effect of modality transition, *F*(1, 21) = 34.2, *p* < .001, η_p_² = .620, indicating longer RTs with a modality switch (752 ms) than with a modality repetition (652 ms). Thus, on average modality-shift costs of 100 ms occurred.

The main effect of CSI, *F*(1, 21) = 90.5, *p* < .001, η_p_² = .812, as well as the interaction of modality transition and CSI were significant, *F*(1, 21) = 29.3, *p* < .001, η_p_² = .582. A long preparation time reduced both the overall RT level (from 776 ms to 627 ms) and the modality-shift costs (from 133 ms to 67 ms). Importantly, none of these effects was influenced by group (*F*s < 1). Additionally, the main effect of group was not significant (*F* < 1).

### Error analysis

The three-way ANOVA with modality transition and CSI as within-subject independent variables and with group as between-subjects variable revealed a significant effect of modality transition, *F*(1, 21) = 22.9, *p* < .001, η_p_² = .522, indicating that a modality switch produced more errors (7.4%) than a modality repetition (4.5%). The effect of CSI was significant, *F*(1, 21) = 5.7, *p* < .05, η_p_² = .212, showing that a long CSI reduced the error rate from 6.5% to 5.3%. The analysis yielded a significant interaction of modality transition and CSI, *F*(1, 21) = 7.1, *p* < .05, η_p_² = .252, indicating that a long CSI reduced modality-shift costs from 4.1% to 1.7%. None of these findings was affected by group (*F*s < 1.5). The main effect of group was not significant (*F* < 1).

#### Effect of response modalities

In a second step, we analyzed each group individually to examine effects of the specific response modalities (vocal vs. manual vs. foot, see Table 2). In the analyses, response modality (vocal vs. manual, vocal vs. foot, manual vs. foot), modality transition (modality switch vs. modality repetition), and CSI (100 ms vs. 1000 ms) were within-subjects independent variables. As we are specifically interested in the influence of response modalities in these analyses, we focus on this variable. However, it is interesting to point out that the main effects of modality transition and CSI as well as the interaction of modality transition and CSI were significant for each group in the individual RT analyses (except the effect of modality transition in the manual/foot group, which was only marginally significant, *F*(1, 21) = 4.9, *p* = .062, η_p_² = .413. Further, the effect of modality transition was also significant in the error analyses of the vocal/foot group, *F*(1, 7) = 14.2, *p* < .01, η_p_² = .669, and the manual/foot group, *F*(1, 7) = 12.3, *p* < .05, η_p_² = .638.

**Table 2.** Experiment 2: Reaction Times as a Function of Modality Transition (Switch vs. Repetition), Response Modality (Vocal vs. Manual, Vocal vs. Foot, Manual vs. Foot), and Cue-Stimulus Interval (100 ms vs. 1000 ms).

	Modality transition		
	Switch	Repetition	Shift costs (switch - repeat)	
Vocal/manual group			
Vocal responses			
CSI 100	1043 (9,0)	894 (6,8)	149 (2,2)
CSI 1000	872 (8,7)	750 (5,9)	122 (2,8)
Manual responses			
CSI 100	753 (5,9)	612 (4,0)	141 (1,9)
CSI 1000	573 (4,9)	521 (4,2)	52 (0,7)
Vocal/foot group			
Vocal responses			
CSI 100	863 (8,4)	735 (2,9)	128 (5,5)
CSI 1000	707 (4,3)	644 (2,8)	63 (1,5)
Foot responses			
CSI 100	760 (8,0)	639 (2,3)	121 (5,7)
CSI 1000	586 (5,0)	551 (3,3)	35 (1,7)
Manual/foot groop			
Manual responses			
CSI 100	819 (9,4)	674 (4,9)	145 (4,5)
CSI 1000	604 (5,3)	534 (4,1)	70 (1,2)
Foot responses			
CSI 100	830 (10,8)	728 (6,5)	102 (4,3)
CSI 1000	628 (8,6)	581 (6,3)	47 (2,3)

*Note*. Reaction times in milliseconds. Error percentage in parentheses. CSI = cue stimulus interval.

With respect to response modality, RT analyses revealed significant main effects of response modality for each group: In the vocal/manual group, RTs were larger for vocal (889 ms) than for manual responses (615 ms), *F*(1, 7) = 55.1, *p* < .001, η_p_² = .887; in the vocal/foot group RTs were larger for vocal (737 ms) than for foot responses (634 ms), *F*(1, 7) = 12.7, *p* < .01, η_p_² = .644; and in the manual/foot group RTs were larger for foot (692 ms) than for manual responses (658 ms), *F*(1, 7) = 8.9, *p* < .05, η_p_² = .559. In the error analyses, the effect of response modality was significant only for the vocal/manual group, *F*(1, 7) = 15.5, *p* < .01, η_p_² = .689, indicating more errors with vocal (7.6%) than with manual (4.8%) responses (for both other groups *F*s < 2.1).

The RT analysis in the vocal/manual group revealed a significant interaction of response modality and modality transition, *F*(1, 7) = 7.4, *p* < .05, η_p_² = .513, showing larger modality-shift costs for vocal (136 ms) than for manual responses (96 ms). Neither the interaction of response modality and CSI, *F*(1, 7) < 2.2, *p* = .18, nor the three-way interaction of response modality, modality transition, and CSI, *F*(1, 7) = 3.1, *p* = .12, were significant. The error analysis in the vocal/manual group yielded no significant interactions (all *F*s < 2.7). Further, neither RT nor error analyses yielded any significant interaction of response modality in the vocal/foot or in the manual/foot group (all *F*s < 1.8).

### Discussion

In Experiment 2, we found modality-shift costs. That is, the performance of subjects was better in modality-repetition trials than in modality-switch trials. Additionally, these modality-shift costs as well as the general RT level were reduced with a long CSI. This finding indicates that subjects could use a long preparation time to prepare for the upcoming response modality. Experiment 2 thus clearly demonstrated that response modalities can play a crucial role in the task-switching paradigm. Furthermore, the data pattern is similar to experiments in which subjects switched between two stimulus categorization tasks (see e.g., Meiran, 1996; Rogers & Monsell, 1995) and thus supports the notion that switching between two response modalities is functionally similar to switching between two judgments.

As an additional finding, the results indicate that it does not matter between which two response modalities subjects have to switch. Furthermore, response modalities did not systematically differ with respect to the size of modality-shift costs. Although modality-shift costs were larger for vocal than for manual responses in the vocal/manual group, we did not observe comparable effects in the other two groups. However, as in Experiment 1, we observed a general influence of the response modality on performance, namely that vocal and foot responses take longer than manual responses. In contrast to Experiment 1, vocal responses in Experiment 2 were even slower than foot responses and resulted in more errors than manual responses.

## General Discussion

In the present study, we examined the role of response modalities in the cognitive representation of tasks by means of a cued task-switching experiment. In Experiment 1, subjects switched between two numerical judgements and the response modality was constant throughout the experiment but manipulated between groups. We observed judgment-shift costs for each response-modality group so that we suppose that the response modality per se does not substantially alter switching between judgments. In Experiment 2, subjects switched between two response modalities and the numerical judgment was constant throughout the experiment. In this experiment, we observed substantial modality-shift costs, indicating the response modalities play a crucial role in task switching.

The main focus of the present study was the effect of switching between response modalities. In contrast to switching between judgments, switching between response modalities requires a flexible adaptation concerning the motor execution of the response. In Experiment 2 of our study, subjects performed the same judgment in each trial but switched between two response modalities to execute a right-left response. The results show costs of modality switching, indicating that “task switching” took place although the judgment was the same in each trial.

The observation that shift costs occur when subjects perform the same numerical judgement in each trial but switch between response modalities constitutes a novel empirical demonstration. This finding is theoretically interesting because it shows the role of response-modality switching when being manipulated in isolation (i.e., independent from a manipulation of judgments or response categories). Additionally, the experiment provides clear evidence that tasks differing only in the response modality are indeed represented as two distinct task sets (see also Philipp & Koch, 2005). As an important consequence, this finding also implies that a “task set” does not include a mapping of a stimulus category to a response category only. Rather the modality in which a response has to be executed appears to be an important information as well. In this way, the present study also suggests that the representation of the response modality is not purely motor-related but plays a role for the cognitive representation of the task. In other words, the response modality does not come into play to simply indicate the result of a “cognitive task” but is an integral part of the cognitive task representation.

In this context, it is also important to point out that we observed no difference for switching between manual and foot responses and switching between vocal and manual or foot responses, respectively. In manual and foot responses, the left/right decision is executed with different effectors, whereas the same effector, namely the mouth, is used for the left/right decision in vocal responses. Thus, response preparation and execution can be assumed to rely on abstract response codes (the words “left” and “right”) in verbal responses, whereas motor preparation and execution is effector-specific in manual and foot responses. Preparing one versus two effectors is also known to influence RTs and neural activity in pointing tasks (see e.g., Adam et al., 2003). Yet, despite the difference in the motor representation, no difference in switching between any combinations of two response modalities was found in our study. This further supports the idea that shift costs observed in Experiment 2 were due to cognitive processes of switching the task and not due to primarily motor-related factors. Additionally, this finding demonstrates that differences in the motor control demand that remain constant across the experiment (i.e., that are manipulated between subjects) do not influence cognitive control processes, replicating the results of the first experiment. Yet, more important, Experiment 2 indicates that motor control processes that are necessary to switch between response modalities, on a behavioral level, cannot be distinguished from and might even be the same as cognitive control processes that are necessary to switch between judgments.

If we understand switching between response modalities as a cognitive process, one might pose the question as to whether switching between two judgments and switching between two response modalities is governed by the same mechanisms and, thus, is functionally similar. Several studies (Allport et al., 1994; Hübner, Futterer, & Steinhauser, 2001; Kleinsorge, Heuer, & Schmidtke, 2004) showed that shift costs and mean RT were comparable when subjects switched between different types of tasks (e.g., switching between judgments and switching between stimulus-response mappings in the study of Kleinsorge et al., 2004). The size of shift costs was also similar between Experiments 1 and 2 of the present study. These observations might tempt one to assume that the same mechanisms are responsible for judgment switching and response-modality switching. Yet, this conclusion would be premature and certainly further research is necessary to indicate whether judgment switching and switching between response modalities is functionally similar or dissimilar.

Apart from the question of whether similar or dissimilar mechanisms govern switching between judgments and switching between response modalities, one can also ask whether the same or different brain areas are activated in both types of task switching. Previous fMRI studies suggested that a frontal-parietal network plays a crucial role in task switching (see e.g., Braver, Reynolds, & Donaldson, 2003; Dove, Pollmann, Schubert, Wiggins, & von Cramon, 2000). As for other tasks that require cognitive control (e.g., Stroop task), the frontal cortex seems to play a major role in switching between tasks (for a meta-analysis, see Derrfuss, Brass, Neumann, & von Cramon, 2005). Yet, these fMRI studies explored switching between judgments only. Thus, it remains an open question as to whether the proposed network is also responsible for switching between response modalities or whether the differences in the nature of the tasks result in the activation of a (partially) different neural network. It is obvious that an answer to this question is necessary to complete our knowledge about the neural mechanisms underlying the flexible adaptation to new situations and to provide a complete (neural) model of task switching.
